# The prevalence of multiple chronic conditions and medical burden in asthma patients

**DOI:** 10.1371/journal.pone.0286004

**Published:** 2023-05-18

**Authors:** Eun-Jung Jo, Young Uk Lee, Ahreum Kim, Hye-Kyung Park, Changhoon Kim

**Affiliations:** 1 Department of Internal Medicine, School of Medicine, Pusan National University, Busan, Korea; 2 Department of Internal Medicine, Pusan National University Hospital, Busan, Korea; 3 Biomedical Research Institute, Pusan National University Hospital, Busan, Korea; 4 Office of Public Healthcare Service, Pusan National University Hospital, Busan, Korea; 5 Department of Preventive Medicine, School of Medicine, Pusan National University, Busan, Korea; University of Buea, CAMEROON

## Abstract

**Background:**

The prevalence of multiple chronic conditions (MCC), defined as several coexisting chronic conditions, has increased with the aging of society. MCC is associated with poor outcomes, but most comorbid diseases in asthma patients have been evaluated as asthma-associated diseases. We investigated the morbidity of coexisting chronic diseases in asthma patients and their medical burdens.

**Methods:**

We analyzed data from the National Health Insurance Service-National Sample Cohort for 2002–2013. We defined MCC with asthma as a group of one or more chronic diseases in addition to asthma. We analyzed 20 chronic conditions, including asthma. Age was categorized into groups 1–5 (< 10, 10–29, 30–44, 45–64, and ≥ 65 years, respectively). The frequency of medical system use and associated costs were analyzed to determine the asthma-related medical burden in patients with MCC.

**Results:**

The prevalence of asthma was 13.01%, and the prevalence of MCC in asthmatic patients was 36.55%. The prevalence of MCC with asthma was higher in females than males and increased with age. The significant comorbidities were hypertension, dyslipidemia, arthritis, and diabetes. Dyslipidemia, arthritis, depression, and osteoporosis were more common in females than males. Hypertension, diabetes, COPD, coronary artery disease, cancer, and hepatitis were more prevalent in males than females. According to age, the most prevalent chronic condition in groups 1 and 2 was depression, dyslipidemia in group 3, and hypertension in groups 4 and 5. Older age, low income, and severe disability were independent risk factors for MCC in patients with asthma. The frequency of asthma-related medical system use and asthma-associated costs increased with increasing numbers of coexisting chronic diseases.

**Conclusion:**

Comorbid chronic diseases in asthma patients differed according to age and sex. The asthma-related-medical burdens were highest in patients with five or more chronic conditions and groups 1 and 5.

## Introduction

A chronic disease is defined as a disease lasting for more than a year and requiring continuous management [1]. The number of patients with chronic diseases is increasing as the population ages, leading the health care system to focus more on long-term health conditions.

The presence of several coexisting chronic conditions in the same individual has been termed “multiple chronic conditions (MCC),” “multimorbidity”, and “comorbidity” [2–4]. However, there is no universally accepted definition of MCC; therefore, estimates of the prevalence of patients with multiple chronic diseases in the general population vary, as 13.1–71.8% [4]. Furthermore, the prevalence of MCC has been increasing with the aging of the population [5].

MCC are associated with poor outcomes, including reduced quality of life, psychosocial problems, high mortality, and high medical costs [6–8]. The MCC working group of the United States Department of Health and Human Services Office of the Assistant Secretary of Health (OASH) selected 20 chronic diseases with a high prevalence that require large investments in public health and clinical interventions [2]. Asthma, one of the chronic diseases selected by the OASH, is a chronic respiratory condition with symptoms indicative of chronic airway inflammation and variable airflow limitations, affecting 1–18% of the general population [2,9]. Asthma is associated with significant morbidity, mortality, and medical costs [10,11]. Many prior studies have focused on asthma-associated comorbidities such as rhinitis, eczema, and obstructive sleep apnea [12,13]. These conditions are associated with poor outcomes, and are generally the focus of physician evaluations. In contrast to asthma-related comorbidities, coexisting chronic diseases in asthma patients have not been studied extensively [14,15]. Few studies have evaluated the prevalence of chronic conditions in asthma patients. In a study based on the National Health and Nutrition Examination Survey in the United States, 54% of adult asthma patients had one or more other chronic conditions [16]. Data from a national telephone interview survey of adults in Germany showed that 61% of asthma patients had one or more other chronic conditions, and the risk of unscheduled inpatient and outpatient asthma care in patients with three or more chronic conditions was significantly higher than that in those with asthma alone [17].

In the present study, we investigated the morbidity of coexisting chronic diseases in asthma patients (including children) and the asthma-related medical burden in patients with MCC (as opposed to asthma-related comorbidities) by investigating morbidity and asthma-related medical costs in this subpopulation.

## Materials and methods

### Study population

The study subjects were recruited from the National Health Insurance Service’s National Sample Cohort (NHIS-NSC). Health insurance is mandatory for all Korean residents, and data related to the use of medical services by individuals are stored by NHIS. NHIS-NSC is a population-based cohort established by the NHIS in the Republic of Korea. It provides information regarding citizens’ utilization of health insurance and health examinations to public health researchers and policymakers based on national records for healthcare utilization and prescriptions [18]. Between 2002 and 2013, the NHIS developed the National Health Information Database (NHID), which records medical bills, treatment details, injury and disease details, and prescription details of all health insurance and medical benefit recipients. The database contains personal, demographic, and treatment data for Korean citizens. Individuals are categorized as insured employees, insured self-employees, and medical aid beneficiaries. Additionally, based on proportional allocation, data from 1 million randomly selected individuals are disclosed to protect personal information. In the present study, we identified asthma patients based on medical histories during 2002–2013 after receiving approval for use of NHIS data.

The following criteria were used to identify asthma patients: 1) International Classification of Diseases, Tenth Revision (ICD-10) codes for asthma (J45.x, J46), 2) use of more than one drug for asthma at least twice per year, including inhaled corticosteroids (ICSs), inhaled long-acting β2-agonists (LABAs), ICS+LABA, inhaled long-acting muscarinic antagonists, inhaled short-acting β2-agonists, inhaled short-acting muscarinic antagonists, theophylline, leukotriene antagonists, systemic corticosteroids, or systemic beta-agonists.

Age was categorized into groups 1–5 (< 10, 10–29, 30–44, 45–64, and ≥ 65 years, respectively). Income level was divided into deciles. Recipients of medical aid were classified into quintiles based on level of aid, with Q5 being the group with the highest income (1st and 2nd as Q1, 3rd and 4th as Q2, 5th and 6th as Q3, 7th and 8th as Q4, and 9th and 10th as Q5). Disability status was classified into three levels defined by the severity index of the Korean disability registration system: no disability, mild disability (grades 3–6), and severe disability (grades 1–2).

### Definition of multiple chronic conditions

We defined MCC as two or more chronic diseases. We analyzed 20 chronic diseases defined by the OASH: arthritis, asthma, autism spectrum disorder, cancer, cardiac arrhythmias, chronic kidney disease, chronic obstructive pulmonary disease (COPD), congestive heart failure, coronary artery disease, dementia, depression, diabetes, hepatitis, human immunodeficiency virus (HIV) infection, hyperlipidemia, hypertension, osteoporosis, schizophrenia, stroke, and substance abuse disorders [2]. The ICD-9-CM codes used to identify OASH chronic diseases were converted to ICD-10 codes using a validated cross-walk algorithm [19]. We used the Chronic Condition Data Warehouse, developed by the Centers for Medicare and Medicaid Services, to identify chronic conditions. Hypertension, hyperlipidemia, diabetes, arthritis, autism spectrum disorder, and osteoporosis were diagnosed if the patient was hospitalized one or more times or received outpatient treatment three or more times with the relevant disease code. Cancer, cardiac arrhythmias, chronic kidney disease, congestive heart failure, coronary disease, artery disease, and stroke were assessed based on one or more outpatient medical records during the 2-year reference period. COPD was defined as ICD-10 codes for COPD or emphysema (J43.0x–J44.x, except for J43.0) as a primary or secondary diagnosis in a subject more than 40 years old.

MCC with asthma was defined if asthma patients extracted from the NHIS-NSC had one or more additional chronic diseases described previously.

### Frequency of asthma-related medical system use and medical costs

The median number of asthma-related contacts with the medical system and the median costs of asthma treatment per year were calculated for each individual to determine the asthma-related medical burden in patients with MCC. We analyzed differences in medical system use by number of coexisting chronic diseases and age group.

### Statistical analysis

Statistical analysis was performed using SAS 9.4. Data are percentages for categorical variables and percentages and 95% confidence intervals (CIs) for the prevalence of comorbid chronic conditions. The prevalence rates of comorbid chronic conditions in all asthma patients and by sex group were age-adjusted using direct standardization. Variables were compared by Pearson’s chi-squared test or Fisher’s exact test for categorical variables. A logistic regression analysis was performed to calculate the odds ratio (OR) and 95% CI. Potentially relevant factors were included in multiple logistic regression analyses to calculate the adjusted odds ratio (aOR). The frequency of asthma-related medical system use and medical costs were analyzed by number of coexisting chronic conditions and age groups using the Kruskal-Wallis test, and presented as medians (interquartile range, IQR). To identify any group differences, we applied the Bonferroni correction method. *P*-values < 0.05 were considered significant.

### Ethics statement

This study was approved by the Institutional Review Board of Pusan National University Hospital (approval no. 1911-001-084). Informed consent was waived because of the retrospective nature of the study.

## Results

### General characteristics of asthma patients

We initially identified 1,125,691 individuals with medical care utilization from the NHID, and the number of asthmatic patients was 146,436. Table 1 shows the characteristics of asthmatic patients using medical care. The prevalence of asthma was 13.01% in the total population, 14.00% in females, and 12.02% in males. According to age group, asthma was most common in group 1 (47.34%), followed by groups 4 and 5. According to income level, asthma was most common in Q4 and Q5 (27.52% and 27.59%, respectively). About 2% of the patients were receiving medical aid, and 3.71% had a disability. Asthma was more prevalent in females than males, and females with asthma tended to be older and poorer than males. The rate of disability was higher in males (Table 1).

**Table 1:** Characteristics of all asthma patients.

	Total (n = 146,436)	Female (n = 78,512)	Male (n = 67,924)	*P*
Age				
< 10	47.34%	40.28%	55.50%	< 0.001
10–29	9.98%	9.57%	10.46%	
30–44	12.19%	15.06%	8.87%	
45–64	17.45%	20.47%	13.96%	
≥ 65	13.04%	14.62%	11.21%	
Income level				
Q5	27.52%	27.00%	28.12%	< 0.001
Q4	27.59%	26.49%	28.87%	
Q3	19.30%	18.85%	19.83%	
Q2	12.74%	13.35%	12.03%	
Q1	10.95%	12.15%	9.56%	
Medical aid	1.90%	2.16%	1.59%	
People with disabilities				
None	96.29%	96.65%	95.89%	< 0.001
Mild	2.98%	2.81%	3.18%	
Severe	0.73%	0.55%	0.94%	
MCC with asthma	36.55%	41.33%	31.02%	< 0.001

### Prevalence of MCC in asthma patients

**Fig 1:**
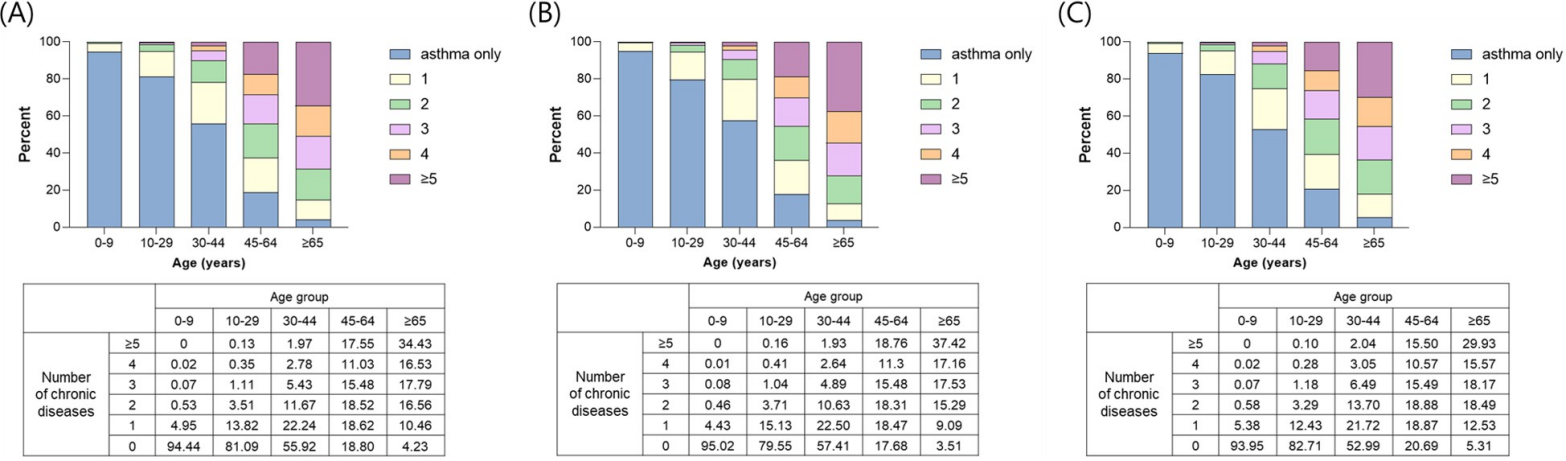
Distribution of prevalence in (A) all, (B) female, and (C) male asthma patients according to the number of coexisting chronic conditions by age group and sex.

The prevalence rates of MCC were 36.55%, 41.33%, and 31.02% in all, female, and male asthma patients, respectively (Table 1); the prevalence was significantly higher in females than males (*P* < 0.001; Table 1). Fig 1 shows the distribution of the prevalence of MCC by age group and sex. The prevalence rates of MCC in asthma patients were 5.56%, 18.91%, 44.08%, 81.20%, and 95.77% in groups 1–5, respectively. The number of chronic diseases tended to increase with age (Fig 1A). The prevalence rates according to age groups for females and males were similar to the overall distribution (Fig 1B and 1C).

Asthmatic females with MCC were older compared to asthmatic males with MCC (Table 2). High income level and severe disability were more common in males than females. Females had a greater number of coexisting chronic diseases than males (Table 2).

**Table 2:** Characteristics of asthma patients with MCC by sex.

	Total (n = 53,521)	Female (n = 32,449)	Male (n = 21,072)	*P*	
Age				
< 10	7.21%	4.85%	10.83%	< 0.001
10–29	5.17%	4.74%	5.83%	
30–44	14.70%	15.52%	13.44%	
45–64	38.77%	40.77%	35.68%	
≥ 65	34.16%	34.12%	34.22%	
Income level				
Q5	29.59%	28.56%	31.19%	< 0.001
Q4	22.42%	21.76%	23.43%	
Q3	16.89%	16.64%	17.27%	
Q2	13.44%	13.81%	12.86%	
Q1	14.48%	15.63%	12.70%	
Medical aid	3.19%	3.60%	2.56%	
People with disabilities				
None	91.25%	92.81%	88.86%	< 0.001
Mild	7.21%	6.16%	8.82%	
Severe	1.54%	1.03%	2.32%	
Number of chronic conditions				
1	30.04%	28.38%	33.05%	< 0.001
2	20.28%	19.66%	21.25%	
3	15.94%	5.96%	15.91%	
4	12.20%	12.73%	11.38%	
≥ 5	21.35%	23.26%	18.41%	

### Characteristics of asthmatic patients with and without MCC

Female sex and older age were more common in asthma patients with MCC than those with asthma only (Table 3). In lower income level groups, it was more common to have MCC with asthma than asthma only (14.48% vs. 8.91% in Q1 and 3.19% vs. 1.15% in the medical aid group; Table 3). MCC with asthma were more common in patients with disabilities (8.75% vs. 0.80%; Table 3).

**Table 3:** Comparison between asthmatic patients with and without MCC.

	MCC with asthma (n = 53,521)	Asthma-only (n = 92,915)	*P*
Female	60.63%	49.58%	< 0.001
Age group			
< 10	7.21%	70.46%	< 0.001
10–29	5.17%	12.76%	
30–44	14.70%	10.74%	
45–64	38.77%	5.17%	
≥ 65	34.16%	0.87%	
Income level			
Q5	29.59%	26.32%	< 0.001
Q4	22.42%	30.58%	
Q3	16.89%	20.70%	
Q2	13.44%	12.34%	
Q1	14.48%	8.91%	
Medical aid	3.19%	1.15%	
People with disabilities			
None	91.25%	99.20%	< 0.001
Mild	7.21%	0.54%	
Severe	1.54%	0.26%	

### Prevalence of coexisting chronic diseases in asthma patients

The age-adjusted prevalence of specific coexisting chronic diseases in asthma patients was as follows (Table 4): hypertension, 23.99%; dyslipidemia, 20.78%; arthritis, 19.71%; diabetes, 13.64%; and COPD, 9.69%. In female asthma patients, the prevalence was as follows: hypertension, 23.61%; arthritis, 22.60%; dyslipidemia, 21.69%; diabetes, 13.17%; and osteoporosis, 10.35%. In male asthma patients, the prevalence was as follows: hypertension, 24.80%; dyslipidemia, 19.52%; arthritis, 14.97%; diabetes, 14.59%; and COPD, 13.52%. Dyslipidemia, arthritis, depression, osteoporosis, congestive heart failure, cardiac arrhythmia, and dementia showed female predominance, whereas hypertension, diabetes, COPD, coronary artery disease, cancer, hepatitis, chronic kidney disease, substance abuse, autism spectrum disorder, and HIV infection showed male predominance.

**Table 4:** Age-adjusted prevalence of comorbid chronic conditions in asthma patients by sex.

	Percent (95% CI)			*P*
	Total (n = 146,436)	Female (n = 78,512)	Male (n = 67,924)	
Hypertension	23.99 (23.69–24.28)	23.61 (23.24–23.98)	24.80 (24.30–25.31)	<0.001
Dyslipidemia	20.78 (20.49–21.08)	21.69 (21.31–22.07)	19.52 (19.04–19.99)	<0.001
Arthritis	19.71 (19.44–19.99)	22.60 (22.23–22.98)	14.97 (14.58–15.37)	<0.001
Diabetes	13.64 (13.42–13.87)	13.17 (12.89–13.46)	14.59 (14.20–14.98)	<0.001
COPD	9.69 (9.51–9.87)	7.35 (7.15–7.55)	13.52 (13.17–13.87)	<0.001
Depression	8.83 (8.63–9.03)	10.15 (9.87–10.42)	6.69 (6.42–6.97)	<0.001
Coronary artery disease	8.50 (8.32–8.67)	8.02 (7.80–8.23)	9.36 (9.06–9.67)	<0.001
Osteoporosis	7.01 (6.85–7.17)	10.35 (10.11–10.59)	1.55 (1.43–1.66)	<0.001
Stroke	5.63 (5.49–5.76)	5.57 (5.39–5.74)	5.72 (5.49–5.95)	0.299
Cancer	5.28 (5.14-–5.42)	4.71 (4.54–4.89)	6.15 (5.91–6.39)	<0.001
Congestive heart failure	4.41 (4.29–4.53)	4.68 (4.53–4.84)	4.00 (3.81–4.19)	<0.001
Hepatitis	3.43 (3.30–3.55)	2.98 (2.83–3.13)	4.22 (3.99–4.45)	<0.001
Chronic kidney disease	3.12 (3.01–3.23)	3.00 (2.86–3.15)	3.31 (3.13–3.49)	0.009
Cardiac arrhythmias	2.59 (2.48–2.69)	2.69 (2.55–2.83)	2.42 (2.26–2.58)	0.014
Dementia	0.77 (0.73–0.82)	0.85 (0.78–0.91)	0.65 (0.58–0.73)	<0.001
Substance abuse	0.73 (0.67–0.79)	0.5 (0.43–0.56)	1.15 (1.03–1.26)	<0.001
Schizophrenia	0.71 (0.65–0.76)	0.7 (0.62–0.77)	0.7 (0.61–0.79)	0.955
Autism spectrum disorder	0.10 (0.07–0.12)	0.04 (0.02–0.05)	0.15 (0.11–0.19)	<0.001
HIV infection	0.02 (0.01–0.02)	0 (0–0.01)	0.04 (0.02–0.06)	<0.001

By age group, the most prevalent chronic condition was depression in groups 1 and 2 (1.14% and 5.33%, respectively), dyslipidemia in group 3 (17.26%), and hypertension in groups 4 and 5 (45.52% and 71.95%, respectively; Table 5).

**Table 5:** Treated prevalence of comorbid chronic conditions in asthma patients according to age.

	Percent (95% CI)					*P*
	< 10 (n = 69,324)	10–29 (n = 14,621)	30–44 (n = 17,850)	45–64 (n = 25,551)	≥ 65 (n = 19,090)	
Hypertension	0.22 (0.19–0.26)	1.96 (1.74–2.19)	12.61 (12.12–13.10)	45.52 (44.91–46.14)	71.95 (71.31–72.59)	< 0.001
Arthritis	0.92 (0.85–0.99)	3.80 (3.49–4.12)	12.26 (11.78–12.75)	36.92 (36.33–37.52)	50.17 (49.46–50.88)	< 0.001
Dyslipidemia	1.03 (0.95–1.10)	5.18 (4.82–5.55)	17.26 (16.71–17.82)	40.83 (40.22–41.43)	35.97 (35.29–36.66)	< 0.001
Diabetes	0.45 (0.40–0.50)	1.98 (1.76–2.22)	7.87 (7.47–8.27)	26.20 (25.66–26.74)	36.28 (35.60–36.97)	< 0.001
COPD	-	-	3.45 (3.19–3.73)	17.64 (17.18–18.12)	36.43 (35.75–37.12)	< 0.001
Coronary artery disease	0.16 (0.13–0.19)	0.87 (0.72–1.03)	4.10 (3.81–4.40)	15.88 (15.44–16.34)	26.38 (25.75–27.01)	< 0.001
Depression	1.14 (1.06–1.22)	5.33 (4.98–5.71)	7.88 (7.49–8.29)	13.11 (12.70–13.53)	15.86 (15.34–16.38)	< 0.001
Osteoporosis	0.01 (0.00–0.02)	0.17 (0.11–0.25)	1.98 (1.78–2.20)	14.16 (13.74–14.59)	23.76 (23.16–24.37)	< 0.001
Stroke	0.12 (0.09–0.15)	0.38 (0.28–0.49)	2.08 (1.87–2.30)	9.42 (9.07–9.79)	21.92 (21.33–22.51)	< 0.001
Cancer	0.20 (0.17–0.24)	0.77 (0.63–0.92)	3.50 (3.24–3.78)	9.07 (8.72–9.43)	15.52 (15.01–16.04)	< 0.001
Congestive heart failure	0.05 (0.03–0.07)	0.18 (0.12–0.27)	1.20 (1.04–1.37)	6.55 (6.25–6.86)	20.40 (19.83–20.98)	< 0.001
Chronic kidney disease	0.67 (0.61–0.74)	1.35 (1.17–1.55)	2.04 (1.84–2.26)	4.87 (4.61–5.14)	7.75 (7.38–8.14)	< 0.001
Hepatitis	0.38 (0.33–0.42)	1.85 (1.64–2.09)	4.13 (3.85–4.44)	5.59 (5.31–5.88)	3.17 (2.93–3.43)	< 0.001
Cardiac arrhythmias	0.34 (0.29–0.38)	1.14 (0.97–1.32)	2.14 (1.93–2.36)	4.04 (3.80–4.28)	5.62 (5.29–5.95)	< 0.001
Dementia	0.04 (0.03–0.06)	0.05 (0.02–0.11)	0.13 (0.09–0.20)	0.99 (0.88–1.12)	4.03 (3.76–4.32)	< 0.001
Schizophrenia	0.11 (0.09–0.14)	0.62 (0.50–0.76)	0.67 (0.55–0.80)	0.76 (0.66–0.88)	1.36 (1.20–1.53)	< 0.001
Substance abuse	0.04 (0.02–0.05)	0.40 (0.31–0.52)	0.94 (0.80–1.09)	1.10 (0.97–1.23)	0.81 (0.69–0.95)	< 0.001
Autism spectrum disorder	0.41 (0.37–0.46)	0.20 (0.13–0.28)	0.00 (0.00–0.02)	0.00 (0.00–0.02)	0.00 (0.00–0.02)	< 0.001
HIV infection	0.01 (0.00–0.01)	0.00 (0.00–0.03)	0.01 (0.00–0.04)	0.04 (0.02–0.08)	0.02 (0.00–0.05)	< 0.001

### Risk factors for MCC in asthma patients

We identified risk factors for MCC in asthma patients by logistic regression analysis (Table 6). Multivariate analysis showed that older age (≥ 45 years), lower income (Q1), and severe disability were independent risk factors for MCC.

**Table 6:** Risk factors for comorbid multiple chronic conditions.

Variable	Univariate analysis		Multivariate analysis	
	OR (95% CI)	*P*	aOR (95% CI)	*P*
Sex (vs. male)				
Female	1.57 (1.54–1.60)	< 0.001	1.02 (0.99–1.05)	0.248
Age (vs. 30–44 years)				
< 10	0.08 (0.07–0.08)	< 0.001	0.08 (0.08–0.08)	< 0.001
10–29	0.30 (0.28–0.31)	< 0.001	0.30 (0.28–0.31)	< 0.001
45–64	5.48 (5.25–5.72)	< 0.001	5.36 (5.13–5.59)	< 0.001
≥ 65	28.75 (26.63–31.03)	< 0.001	27.39 (25.36–29.59)	< 0.001
Income level (vs. Q5)				
Q4	0.65 (0.63–0.67)	< 0.001	0.99 (0.95–1.04)	0.997
Q3	0.73 (0.70–0.75)	< 0.001	1.01 (0.96–1.06)	0.417
Q2	0.97 (0.94–1.00)	< 0.001	1.01 (0.96–1.07)	0.358
Q1	1.45 (1.39–1.50)	< 0.001	1.06 (1.01–1.12)	0.003
Medical aid	2.46 (2.27–2.66)	< 0.001	0.89 (0.78–1.01)	0.035
Disability (vs. none)				
Mild	14.41 (13.12–15.81)	< 0.001	1.79 (1.60–1.99)	0.319
Severe	6.43 (5.57–7.42)	< 0.001	3.71 (3.02–4.55)	< 0.001

### Asthma-related medical burden in asthma patients with MCC

The frequency of asthma-related medical system use increased significantly with the number of coexisting chronic diseases (Table 7). The median frequency of asthma-related medical system use was higher in the group with five or more chronic diseases than in the asthma-only group. Asthma-related medical costs also increased with the number of coexisting chronic diseases (Table 7). The median was higher in patients with five or more chronic diseases than in asthma-only patients.

**Table 7:** Comparison of annual asthma-related medical system use and costs in asthma patients by number of comorbid diseases.

Number of chronic diseases	Number of patients	Medical system use (number of times)		Medical costs	
		Median (IQR)	*P*	Median (IQR)	*P*
0	92,915	2.50 (2.00–4.00)*^†^	< 0.001	60.40 (34.88–119.87)*	< 0.001
1	16,173	2.00 (2.00–3.33)		54.25 (31.66–107.77)^†^	
2	10,856	2.33 (2.00–3.67)		54.75 (31.54–106.24)^†^	
3	8,533	2.50 (2.00–4.00)*^‡^		57.77 (33.08–114.40)	
4	6,531	2.50 (2.00–4.00)^†^^‡^		62.59 (35.35–126.15)*	
≥ 5	11,428	2.75 (2.00–4.00)		79.73 (42.61–160.43)	

*^†‡^In the post hoc analysis, there was no significant difference between the two groups. For the other groups, a significant difference was observed between each group (*P* < 0.05).

Unit of medical costs: Thousand Korean won.

In our analysis of age, group 5 showed the most frequent medical system use, followed by group 1 (Table 8). Group 1 had the highest medical costs, followed by group 5.

**Table 8:** Comparison of annual asthma-related medical system use and costs in asthma patients with multiple chronic conditions by age group.

Age group	Number of patients	Medical system use (number of times)		Medical costs	
		Median (IQR)	*P*	Median (IQR)	*P*
< 10	3,857	2.50 (2.00–3.67)*	< 0.001	83.54 (44.37–163.19)	< 0.001
10–29	2,765	2.00 (2.00–3.00)		51.28 (30.45–99.42)*	
30–44	7,868	2.00 (2.00–3.00)		52.53 (31.29–100.83)*	
45–64	20,748	2.33 (2.00–3.60)*		58.13 (33.06–115.01)	
≥ 65	18,283	3.00 (2.00–4.25)		65.03 (35.53–133.54)	

*In the post hoc analysis, there was no significant difference between the two groups. For the other groups, a significant difference was observed between each group (*P* < 0.05).

Unit of medical costs: Thousand Korean won.

## Discussion

We examined the frequency and characteristics of coexisting chronic conditions in asthma patients and their medical burdens. The prevalence of MCC in asthmatic patients was 36.55% overall, higher in females, and increased with age. The most frequent coexisting chronic diseases were hypertension, dyslipidemia, arthritis, and diabetes, but distribution differed according to sex and age. Old age, low income, and severe disability were independent risk factors for MCC. The frequency of asthma-related medical system use and costs increased with the number of chronic diseases and were higher in patients with five or more chronic diseases than in asthma-only patients. Children and older adults used medical care more frequently and had higher medical costs than other age groups.

There are various estimates of the prevalence of MCC in asthma patients: 54% in the United States (2,873 asthma patients, ≥ 20 years of age, mean age 44.5 years, 58.0% female) [16], 61.1% in Germany (2,242 asthma patients, ≥ 18 years of age, mean age 52.5 years, 58.6% female) [17], and 59% in Canada (10,089 asthma patients, ≥ 18 years of age, 64.2% female) [20]. In our study, the prevalence of MCC with asthma was 36.55%, which was lower than that reported previously. The difference in prevalence is probably attributable to differences in our sample or the definitions of MCC. We included all age groups (adults and children) and defined MCC in asthma patients as the presence of one or more chronic diseases from the OASH list in addition to asthma. Despite differences in the study population and prevalence of chronic diseases in previous studies, the common coexisting chronic diseases were similar [16,17,20,21].

Almost all studies of comorbidities in asthma patients have focused on adults. The presence of MCC is considered common among older adults. In the present study, the common coexisting diseases among older adult asthma patients reflected the overall prevalence of chronic diseases. However, we investigated chronic diseases in asthma patients of all ages. We found that MCC occurred even in children and young adults and identified age-based differences in coexisting chronic diseases. Children and young adults with asthma had depression more often than other chronic diseases. Mental illness, including depression, was reported as a major comorbidity in asthma patients [17,20]. A study using the Veterans Affairs healthcare national database reported a varying prevalence of depression according to age [22]. Depression was the most prevalent chronic condition in 18- to 45-year-old asthma patients [22]. A Canadian study of adult asthma patients reported that the most common chronic condition in young adults (18–45 years old) was depression [23]. Anxiety and mood disorders are associated with poor asthma control and greater health service use in children and adolescents [24,25], as well as poorer adherence to asthma therapy and asthma outcomes [26,27]. We included children with asthma in our analysis and found that depression was more prevalent than other diseases. The most prevalent coexisting chronic disease in each age group differed from prevalence in the general population, with particularly notable differences in young asthma patients. Therefore, age-based evaluation and management of common chronic diseases is critical to improving health outcomes.

We identified common chronic diseases by sex. The prevalence of MCC was higher in females overall, as well as in female asthma patients [16,17,20]. COPD, coronary artery disease, cancer, hepatitis, substance abuse, autism spectrum disorder, and HIV infection were more prevalent in males. These trends may be related to the higher frequency of smoking and alcohol consumption in males [28,29], suggesting the importance of lifestyle and environmental interventions.

Risk factors for the presence of MCC were old age, low income level, and severe disability. Multivariate analysis showed that female sex was not a risk factor, despite a higher prevalence of MCC among females. Previous studies have reported a negative association between higher income and progression of MCC [30,31], as well as a high frequency of medication non-adherence in patients of low income [32]. This may explain the high risk of MCC in the low-income group.

The presence of MCC influences healthcare use, costs, and mortality [22,33]. In the present study, the asthma-related medical burden increased with the number of coexisting chronic conditions. Furthermore, patients with five or more chronic conditions had a much greater asthma-related medical burden. The presence of several chronic conditions leads to attendance at multiple medical institutions, which creates a risk of exposure to duplicate or incompatible treatments. For this reason, individuals with MCC may experience worse outcomes than individuals with a single chronic condition. The medical burden increased in age groups 1 and 5, indicating that asthma is more severe in children and older adults than in other age groups. In the present study, many older asthma patients had several additional chronic diseases. However, although children had a lower prevalence of MCC compared other groups, their asthma-related medical burden was higher than that of adults. We believe that physicians should be concerned about asthma management in patients with MCC and should monitor these patients to reduce the morbidity associated with multiple chronic diseases.

This study had several limitations. First, this study included a randomly selected representative sample. Non-citizens and employees with an unidentifiable income level were excluded from the database, which may have led to selection bias. However, Lee et al. [18] have shown that the cohort database is representative of the population in terms of residence area, insurance premium, and prevalence of major diseases. Second, we defined asthma according to diagnostic code, not diagnostic examination. However, other studies using NHIS-NSC data also defined asthma in this way [34,35]. Third, the prevalence of chronic diseases in this study was based on diagnostic codes. Therefore, we may have underestimated their prevalence. For example, depression was identified in 8.83% of our sample, but there is reportedly a treatment gap in mental disorders in Korea [36]. In addition, we used the OASH list to evaluate coexisting chronic conditions. Comorbidities may have different distributions depending on the country. However, the major comorbidities in asthma patients in our study were similar to those reported elsewhere. This suggests that common chronic diseases are similar despite racial differences. Furthermore, we determined that depression was more prevalent than other diseases in children and young adults even though it was not one of the most common diseases in the overall sample. Therefore, one of the strengths of our study is its inclusion of patients of all ages and its analysis of diseases that require public health and clinical interventions.

## Conclusions

We determined that coexisting chronic diseases in asthma patients differed by age and sex. Older asthma patients of low economic status were more vulnerable to MCC. The asthma burden increased with the addition of more chronic diseases. Therefore, physicians providing asthma management should be aware of coexisting chronic diseases in asthma patients and endeavor to reduce morbidities in vulnerable patients.
